# Mass Spectrometry Approaches for Identification and Quantitation of Therapeutic Monoclonal Antibodies in the Clinical Laboratory

**DOI:** 10.1128/CVI.00545-16

**Published:** 2017-05-05

**Authors:** Paula M. Ladwig, David R. Barnidge, Maria A. V. Willrich

**Affiliations:** Department of Laboratory Medicine and Pathology, Mayo Clinic, Rochester, Minnesota, USA; UMKC School of Medicine

**Keywords:** therapeutic monoclonal antibodies, mass spectrometry, proteomics, tryptic peptides, intact light chain mass, clinical laboratory, immunogenicity, therapeutic drug monitoring, immunotherapy

## Abstract

Therapeutic monoclonal antibodies (MAbs) are an important class of drugs used to treat diseases ranging from autoimmune disorders to B cell lymphomas to other rare conditions thought to be untreatable in the past. Many advances have been made in the characterization of immunoglobulins as a result of pharmaceutical companies investing in technologies that allow them to better understand MAbs during the development phase. Mass spectrometry is one of the new advancements utilized extensively by pharma to analyze MAbs and is now beginning to be applied in the clinical laboratory setting. The rise in the use of therapeutic MAbs has opened up new challenges for the development of assays for monitoring this class of drugs. MAbs are larger and more complex than typical small-molecule therapeutic drugs routinely analyzed by mass spectrometry. In addition, they must be quantified in samples that contain endogenous immunoglobulins with nearly identical structures. In contrast to an enzyme-linked immunosorbent assay (ELISA) for quantifying MAbs, mass spectrometry-based assays do not rely on MAb-specific reagents such as recombinant antigens and/or anti-idiotypic antibodies, and time for development is usually shorter. Furthermore, using molecular mass as a measurement tool provides increased specificity since it is a first-order principle unique to each MAb. This enables rapid quantification of MAbs and multiplexing. This review describes how mass spectrometry can become an important tool for clinical chemists and especially immunologists, who are starting to develop assays for MAbs in the clinical laboratory and are considering mass spectrometry as a versatile platform for the task.

## INTRODUCTION

Monoclonal antibody (MAb) therapy is a relatively novel, growing field in the pharmaceutical industry, with an expected $100 billion dollars in sales across the world in 2017. As more patients undergo MAb therapy, there is a perceived need for monitoring of MAb therapeutic efficacy and understanding loss of response when patients fail treatment. This poses an opportunity for the clinical laboratory to develop a new niche of tests and improve patient care by personalizing therapeutic regimens with MAbs.

Therapeutic MAbs are typically modeled after human immunoglobulins (Igs), homodimers with a molecular mass of approximately 150 kDa. Each Ig consists of two identical glycosylated heavy chains (50 to 70 kDa) and two identical light chains (22 to 24 kDa). The light chains are linked to the heavy chains by a single disulfide bond, while the heavy chains are linked together by two or more disulfide bonds, depending on the Ig isotype and/or subclass. While the N-terminal portion of both the light and heavy chains contains the variable-region sequences that give the Ig its specificity for binding of antigens (Fab), the C-terminal portion of both chains (Fc) contains the constant-region sequences that characterize the Ig by isotype and class (1, 2). It is critical to understand the relationship between the constant region and the variable region of Igs and therefore therapeutic MAbs.

When using mass spectrometry (MS) to characterize and quantify MAbs, the variable region is the target and its uniqueness is compared to the endogenous polyclonal Ig repertoire. The conserved amino acid sequences designate the heavy chain isotype IgG, IgA, IgM, IgD, or IgE and the light chain either kappa or lambda. The IgG isotype has four subclasses with different characteristics: IgG1, IgG2, IgG3, and IgG4. Therapeutic MAbs currently on the market are predominately IgG1 kappa, with a few being IgG2, IgG4, or hybrids.

## MAb PRODUCTION AND TARGETS

International nonproprietary names (INNs) have been assigned to MAbs since 1991 and use -MAb as a stem and specific substems to cover their source (-omab for murine, -ximab for chimeric, -zumab for humanized, and -umab for human antibodies). In 2014, the World Health Organization proposed a new nomenclature system because of the growth of the field and the number of techniques for engineering and manufacturing MAbs. Although the proposal has generated much controversy, since it would change the classification of the MAbs currently on the market (3), a new naming system is still under discussion. Using the 1991 system, the variable region of chimeric MAbs is from a nonhuman source, usually murine, and linked by disulfide bonds to a human Ig. In humanized MAbs, only the variable light- and heavy-chain regions that bind antibody, or complementarity-determining regions, are nonhuman and therefore “grafted” onto human variable regions. Fully human MAbs are produced by using transgenic mice and phage display techniques (4). MAb production involves a challenging combination of recombinant expression technology and purification techniques (5).

MAbs work by blocking targeted molecule functions, inducing apoptosis of cells that express the target, or modulating signaling pathways. Targets may be membrane bound, soluble, or both. Most MAbs are used in oncology (∼38%), autoimmune/inflammatory conditions (∼27%), and neurology areas (∼12%). Successful targets in oncology include CD20 (rituximab [RTX]), CD38 (daratumumab [DARA]), and programmed-death ligand 1 (PD-L1) or programmed-death receptor 1 (PD-1) immune checkpoint inhibitors (pembrolizumab [PEMBRO] and others). For autoimmune disorders, there are several tumor necrosis factor alpha (TNF-α) inhibitors (infliximab [IFX], adalimumab [ADM], and others) and anti-interleukin-12 (IL-12)/IL-23 (ustekinumab) and other cytokine inhibitors. In rare diseases and neuroscience, the use of less obvious targets such as the complement C5 inhibitor eculizumab (ECU) and natalizumab (NATA), which targets the cell adhesion molecule α4-integrin, have made it to the clinic (Table 1).

**TABLE 1:** Examples of therapeutic MAbs mentioned in this review^*a*^

INN	Brand name(s)	Target; format	Indication(s) first approved or reviewed	1st U.S. approval yr
Oncology				
Daratumumab	Darzalex	CD38; human IgG1	Multiple myeloma	2015
Elotuzumab	Empliciti	SLAMF7; humanized IgG1	Multiple myeloma	2015
Nivolumab	Opdivo	PD1; human IgG4	Melanoma, non-small-cell lung cancer	2014
Pembrolizumab	Keytruda	PD1; humanized IgG4	Melanoma	2014
Siltuximab	Sylvant	IL-6; chimeric IgG1	Castleman's disease	2014
Trastuzumab	Herceptin	HER2; humanized IgG1	Breast cancer	1998
Rituximab	MabThera, Rituxan	CD20; chimeric IgG1	Non-Hodgkin lymphoma	1997
Autoimmune/immunology				
Vedolizumab	Entyvio	α4β7 integrin; humanized IgG1	Ulcerative colitis, Crohn's disease	2014
Ustekinumab	Stelara	IL-12/23; human IgG1	Psoriasis	2009
Certolizumab pegol	Cimzia	TNF-α; humanized Fab, pegylated	Crohn's disease	2008
Adalimumab	Humira, Amjevita	TNF-α; human IgG1	Rheumatoid arthritis	2002
Infliximab	Remicade, Inflectra	TNF-α; chimeric IgG1	Inflammatory bowel disease, rheumatoid arthritis	1998
Rare diseases/neurology				
Eculizumab	Soliris	C5; humanized IgG2/4	Paroxysmal nocturnal hemoglobinuria, atypical hemolytic-uremic syndrome	2007
Natalizumab	Tysabri	α4 integrin; humanized IgG4	Multiple sclerosis	2004

a This list is not meant to be exhaustive. Currently, there are over 60 FDA-approved MAbs. A complete and frequently updated list of approved MAbs can be found online at www.antibodysociety.org.

## MAb METABOLISM

Therapeutic MAbs have unique characteristics compared to small-molecule drugs. Their metabolism does not undergo traditional hepatic cytochrome P450 oxidation, phase II enzyme glucuronidation, or phase III elimination via cellular transporters. Instead, MAbs are closer to human Igs, which are synthesized in the rough endoplasmic reticulum of plasma cells and secreted into the extracellular space. The Fc portion of the Ig is recognized by Fc receptors (FcγRs) on the surface of endothelial cells. FcγR1 and FcγR2 internalize the Igs, leading to lysosomal degradation. In contrast, the neonatal Fc receptor (FcRn) recycles the Ig through endosomes with subsequent secretion back into circulation (6). The pharmaceutical industry has invested in several modifications (Fc engineering) to increase MAb half-lives in circulation in order to increase efficacy. Most MAbs in clinical use have half-lives of a week or longer, resulting in long dosing intervals (2 to 8 weeks). Intravenous administration allows for application of larger volumes, lower immunogenicity, and higher bioavailability, whereas more convenient subcutaneous injections work well for smaller volumes.

## WHY MEASURE THERAPEUTIC MAbs IN THE CLINICAL LABORATORY

Therapeutic MAb use has expanded significantly in the last 5 years, and depending on their target or concentration, MAbs may impact the routine clinical testing of patients. The first report of such interference was published in 2010, when serum protein electrophoresis (SPEP) and immunofixation (IFE) were ordered on a multiple myeloma patient on MAb therapy with siltuximab, targeting IL-6 (7). RTX and vedolizumab (VEDO) are also visualized as a fuzzy small band on SPEP when measured at their peak, 1 to 2 days after MAb infusion, and an IgG kappa is identified in the gamma fraction on IFE (8). SPEP and IFE are ordered for a number of indications, including autoimmune/infectious diseases (polyclonal hypergammaglobulinemia), renal dysfunction (nephrotic proteinuria), and primary immunodeficiency (hypogammaglobulinemia), diseases for which treatment with a therapeutic MAb is becoming common. These tests are also the most important part of the diagnostic workup for patients with suspected plasma cell dyscrasias, and a monoclonal protein report is a finding that may trigger additional investigations, such as bone marrow, kidney, or heart biopsies. Methods to overcome this interference in the laboratory have been published but are not yet widely available. MS-based methods have the potential to be utilized (8–10), and incubation with anti-idiotypic antibodies against the therapeutic MAb changes its electrophoretic migration, allowing it to be discriminated from the original endogenous clone when they comigrate by SPEP and IFE (11). Immunoassays may also be impacted by the presence of therapies such as DARA, which causes panreactivity in antibody screenings in transfusion medicine, as recently reported by blood banking services (12).

The use of the term companion diagnostics has gained popularity and has been used liberally; however, the FDA defines it as an *in vitro* diagnostic device or imaging tool that provides essential information for the safe and effective use of a corresponding therapeutic product. Its use is stipulated in the instructions for use in both labels, the *in* *vitro* diagnostic test and the therapeutic product. Companion diagnostics are used before prescribing an oncology MAb (e.g., HER2-positive tissue biopsy before prescription of trastuzumab for metastatic breast cancer or the presence of PD-1/PD-L1 before prescription of PEMBRO or nivolumab (NIVO) for non-small-cell lung cancer); however, once therapy is indicated, MAbs are used in high doses and usually for a defined amount of time. Therapeutic efficacy is measured as tumor shrinkage and based on clinical symptoms, not necessarily therapeutic drug monitoring through laboratory testing. On the other hand, in autoimmune disorders, the duration of MAb therapy is expected to be lifelong.

In inflammatory bowel disease (IBD), a chronic autoimmune group of disorders characterized by severe inflammation of different layers of the gastrointestinal tract, the introduction of MAbs targeting TNF-α was a game changer that significantly improved the quality of life for patients, delaying the onset of abdominal surgeries. Optimizing therapy during the induction phase so that IFX or ADM concentrations are kept above certain thresholds has proven to be associated with improved responses and better outcomes (13) in IBD. Despite their overall therapeutic efficacy, more than one-third of patients on TNF-α inhibitors show no response to induction therapy (primary nonresponders) and in up to 50% of the responders, therapy becomes ineffective over time (secondary nonresponders) (14–16). Reasons for primary loss of response include disease processes mediated by proinflammatory molecules other than TNF-α. Secondary loss of response is associated with low albumin, a high body mass index, the degree of systemic inflammation and immune response to therapy, or immunogenicity (6).

Immunogenicity, the development of an immune response to the MAb, with appearance of antidrug antibodies (ADAs), can significantly hinder the efficacy of MAb therapy, especially when the ADAs formed neutralize its effect. Laboratory testing of patients for quantitation of the MAb concentrations in circulation and assessment of immunogenicity can help optimize therapy when a partial response or a loss of response to therapy is observed. Results from laboratory testing play an important role in patient management, considering the evidence accrued for the TNF-α inhibitor class in IBD (13, 14, 17–20). When trough levels are measured in patients who have undetectable or low concentrations of the MAb but no detectable ADAs, the physician may choose to increase the dose of the MAb in an attempt to increase the amount of the drug in circulation. If the patient has a low MAb concentration in the presence of ADAs, the physician may switch the patient to another MAb in the same class, or it may be necessary to switch the patient to therapy with a different target (15). For other MAbs outside the IBD setting, comprehensive studies showing the links among MAb concentrations, loss of response, and outcomes are not available and the recommendations for patient management based on serum monitoring of MAb concentrations is less clear, although it seems that the TNF-α inhibitor experience can be translated to other areas (21, 22).

In summary, there are two main needs for measuring therapeutic MAbs in the clinical laboratory setting, (i) to differentiate the drug from an endogenous monoclonal protein and prevent additional testing or reporting of erroneous results and (ii) to develop assays to measure MAb concentrations and immunogenicity to aid in the monitoring of therapeutic efficacy and loss of response.

## METHODOLOGIES AVAILABLE FOR MAb MEASUREMENT IN THE CLINICAL LABORATORY

Clinical tests for IFX, ADM, RTX, certolizumab (CERT), VEDO, and ECU are available commercially in the United States (Table 2). For IFX and ADM, which are among the top five best-selling MAbs, measurement of trough serum MAb concentrations is increasingly becoming part of routine patient management. Methodologies for measurement of IFX and ADM vary widely, ranging from traditional ELISAs (23, 24), passing on to mobility shift high-performance liquid chromatography (LC) (25), to cell-based assays with a luciferase reporter gene (26, 27). The assays are offered as either panels or reflex tests with MAb quantitation and measurement of the presence of ADAs. Immunogenicity is invariably assessed by using immunoassays because of its heterogeneous nature, and the caveats associated with those methods have been recently reviewed (26). MAb quantitation may be performed with immunoassays, cell-based assays, LC, or MS. Last but not least, MS approaches to MAb quantitation, used extensively in the development of MAbs by pharmaceutical industries, have entered the clinical laboratory and will be described in detail.

**TABLE 2:** MAb methodologies used in this study^*a*^

Method	IFX	ADM	RTX	CERT	VEDO	ECU
Non-MS-based methods						
Cell-based reporter gene assay	X (27)	X (27)				
ELISA	X	X		X	X	X
Electrochemiluminescence immunoassay	X	X	X			
Homogeneous mobility shift assay	X (25)	X (64)			X	
MS-based methods						
Tryptic digest by LC-MS/MS	X (59)	Lack of unique tryptic peptide sequences; peptides found in a few healthy subjects not taking drug at clinically relevant concn	Not studied	Not studied	Method developed and quantitation of VEDO possible with unique light chain tryptic peptides (data not shown)	Quantitation of ECU possible with unique light-chain tryptic peptides (data not shown)
Intact light chain by LC-MS	In initial feasibility studies, did not meet required LOQ with Melon Gel as preanalytical enrichment method	In initial feasibility studies, did not meet required LOQ with Melon Gel as preanalytical enrichment method	RTX light chain characterized and measured in a series of samples; however, clinical utility remains to be shown (61)	Not studied	VEDO light chain characterized with Melon Gel as preanalytical enrichment method (data not shown)	Quantitation achieved by IgG4 preanalytical sample enrichment and intact ECU light-chain accurate mass (63)

a X, commercially available in CLIA-certified laboratories. Reference numbers are in parentheses. Comments are from the authors' experience with MS methods.

## HISTORY OF MAb MEASUREMENT BY MS

Not long after electrospray ionization (ESI) and matrix-assisted laser desorption ionization (MALDI) were introduced commercially in the early 1990s as a means of transferring proteins into the gas phase, mass spectrometrists working in the pharmaceutical industry began to explore the structure of MAbs by using MS (28). Much of the early work combined LC with MS to characterize therapeutic MAbs as recombinant protein expression technology made the widespread use of MAbs a reality (29–31). The ability of MS to rapidly provide information on posttranslational modifications (PTMs) in this complex class of drugs soon made it the technique of choice in pharmaceutical research labs (32, 33). By the early 2000s, mass spectrometrists were discovering that absolute quantification could be achieved by combining protease specificity with stable-isotope-labeled (SIL) internal standard (IS) peptides with triple-quadrupole mass spectrometers that are routinely used in clinical laboratories (34–36). Soon after, pharmaceutical laboratories began to use the same MS methods instead of ELISAs to quantify MAbs in selected primate studies (37, 38). As new mass spectrometers with higher resolution and improved mass measurement accuracy became available around 2005, pharma began to routinely investigate PTMs such as methionine oxidation and glycosylation, which could be easily identified in Fc and Fab fragments by simply observing mass shifts between protein species (39). At the same time, novel fragmentation methods were being developed that allowed extensive characterization of MAbs in the gas phase (40–42). Currently, MS plays a central role in the routine quality control of therapeutic-MAb production (43–45), and new MS-based methodologies for monitoring are continuously appearing in the literature. The remainder of this review will focus on translating MS-based methods for quantifying therapeutic MAbs into the clinical laboratory with a focus on selecting methods that result in the highest level of analytical sensitivity and specificity with respect to each patient's endogenous Ig repertoire.

## MS TECHNIQUES USED FOR MAb QUANTIFICATION IN THE CLINICAL LABORATORY

**FIG 1:**
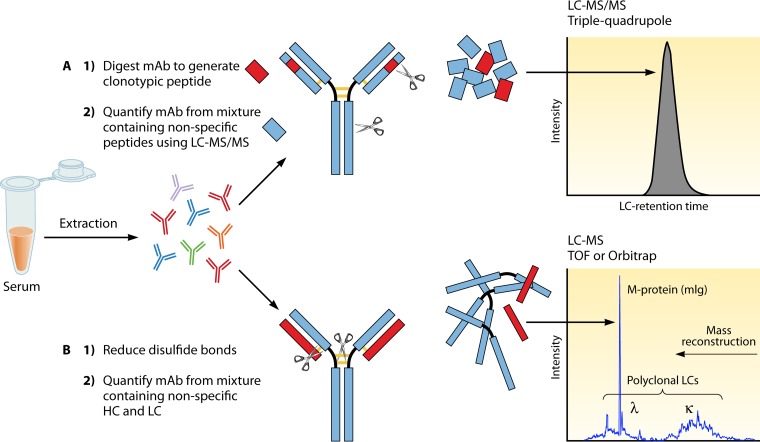
LC-MS versus LC-MS/MS for quantitation of therapeutic MAbs. MAbs can be quantitated by MS. Ig extraction or enrichment techniques (protein crash, Melon Gel IgG enrichment, or affinity matrix for specific IgG subclasses, for example) will help reduce the protein load in the sample. (A) LC-MS/MS method. The LC-MS/MS method includes separation of the light chains from the heavy chains after reduction of the disulfide bonds, followed by trypsin cleavage to generate multiple peptides from the intact Ig. Peptides specific to the variable region of the MAb on either the light or the heavy chains, which do not cross-react with human sequences, are used to quantitate the MAb. Serum samples are extracted/enriched to reduce the protein load. Samples are denatured (protein is unfolded), reduced (cysteine reduction breaks the disulfide bonds connecting MAb light and heavy chains), alkylated (alkylation of cysteines prevents the disulfide bonds from reforming), and digested by trypsin into smaller peptides. The peptide mixture is separated by LC before analysis by MS/MS. The mass of the peptide of interest (parent ion) is recorded, the peptide is further cleaved inside the mass spectrometer (fragment ion), and the ion pair transition is utilized for quantitation, preferably on triple-quadrupole instruments. (B) LC-MS method. The LC-MS method separates the light chains (LC) from the heavy chains (HC) through reduction, and the intact light chains are not further processed or cleaved. Instead, their accurate mass is measured. In healthy individuals, the polyclonal repertoire of lambda and kappa light chains follows a Gaussian distribution at a 1:2 lambda-to-kappa ratio, and MAbs present in significant amounts stand out of the polyclonal endogenous background as peaks or spikes.

LC-MS and LC-MS/MS (tandem MS) are terms related to MS-based techniques used to analyze therapeutic MAbs (Fig. 1). For both acronyms, the LC portion is the same; a liquid chromatograph is used to perform LC where analytes are separated on a column prior to ionization. The MS portion of the acronym relates to the form the analyte is in when detected in the mass spectrometer. If LC-MS is being performed, then the intact analyte is being analyzed (e.g., for MAbs, intact light chain). If LC-MS/MS is being performed, then a fragment of the analyte is being analyzed (e.g., tryptic peptides of either light- or heavy-chain variable regions). It is important to note that both acronyms leave out an additional acronym, ESI. It is now assumed that ESI is being used to generate ions when a liquid chromatograph is coupled to a mass spectrometer for protein analysis. It is important to keep in mind that no matter what mass spectrometer is being used, gas phase analyte ions must be formed first in order for the mass spectrometer to detect them. Furthermore, if the ions are formed at atmospheric pressure, as is the case with ESI, then the ions have to be transferred into the mass spectrometer, which is under vacuum and at a much lower pressure (for excellent reviews, see references 46 and 47).

The most commonly used mass spectrometer in the clinical laboratory for both the LC-MS and LC-MS/MS methods is the triple-quadrupole mass spectrometer (Table 3). Triple-quadrupole mass spectrometers have been the mainstay of clinical laboratories performing forensic toxicology since the late 1990s and continue to be the instrument platform of choice for clinical labs (48, 49). They are extremely sensitive, specific, stable instruments and have a proven track record of providing precise and accurate quantitative results. Triple-quadrupole mass spectrometers also have the greatest linear dynamic range for quantifying analytes of any class and are ideally suited for quantifying peptides produced after trypsin digestion of proteins, such as the ones generated from MAbs.

**TABLE 3:** Main differences between MS methods for MAb analysis

Main characteristic	MS/MS of proteotypic or clonotypic peptides specific to variable region of MAb with triple-quadrupole mass spectrometer^*a*^	Intact-protein mass measurement by MS		
		MALDI-TOF^*b*^	Q-TOF^*b*^	Orbitrap^*b*^
Instrument cost	$400,000	$150,000	$300,000	$500,000
Common applications in the clinical lab	Toxicology, endocrinology quantitative assays	Microbiology, bacterial identification	Toxicology drug screening	Mainly present in research labs and esoteric reference laboratories
Applications for MAbs	Quantitation of MAbs	Screening of therapeutic MAb	Characterization and quantitation of multiple MAbs	High-resolution characterization and quantitation of multiple MAbs

a Labor-intensive, includes reduction, alkylation, and trypsin digestion.

b Melon Gel enriches for all IgGs. LOQ of MAb quantitation and removal of MAb target should be considered. IgG4 capture is an enrichment option for IgG4 MAbs.

Other instruments that are used to quantify MAbs by LC-MS and LC-MS/MS include time of flight (TOF) and Orbitrap mass spectrometers. These instruments have mass measurement accuracy, resolution, and scanning speed superior to those of triple-quadrupole mass spectrometers, making them ideal for analyzing intact MAb light and heavy chains. TOF and Orbitrap mass spectrometers have only recently been utilized for quantitative LC-MS and LC-MS/MS experiments in the clinical laboratory, so their track record is not yet well established. Fortunately, the LC systems and ESI sources used for all three classes of instruments are virtually identical; therefore, the primary performance factors separating them are sensitivity and robustness. In summary, triple-quadrupole mass spectrometers are currently preferred for quantifying tryptic fragments of MAbs while TOF and Orbitrap instruments are preferred for quantifying intact light and heavy chains.

## TRANSLATING METHODS TO THE CLINICAL LABORATORY

In translating a new method into the clinical laboratory, especially for the ones that do not have FDA-approved status and are considered laboratory-developed tests (LDTs), as are all MAb methods at this time, it is important that the method has been firmly established and proved robust by using an assay life cycle model (50). There are two stages of method development, (i) establishment, in which the method is developed and validated, and (ii) implementation, in which the clinical laboratory verifies the robustness of the method. The steps in the establishment stage of an assay life cycle can consist of feasibility and design, development, and validation. Documentation is an integral part of method development. Beyond the certification of the performance of a method, there should also be predetermined decision points at each step on whether acceptance criteria have been met for that particular step and whether it is feasible to move on to the next step. In feasibility and design, a literature review is performed, the clinical usefulness (intended use) is determined, the legal right to use the developed method is obtained, and a marketing assessment is performed. Lastly, a feasibility evaluation should be performed to determine if the knowledge, equipment, and resources needed to move forward are available.

In the development step, a method is transitioned from concept to reality through iterative improvements of instrumentation, reagents, calibrators, controls, and experimental steps. The final products of the development step would be a standard operating procedure (SOP) along with preliminary experimental data showing that, for example, the needs of the method in the form of analytical sensitivity or the limit of quantitation (LOQ) are consistent with the therapeutic concentrations observed in the population and initial imprecision estimates. The last step of the establishment stage is validation. In validation, a locked SOP is followed to perform critical experiments outlined in a validation plan to meet specific acceptance criteria. Critical experiments would include, but not be limited to, experiments to determine within-run and within-laboratory imprecision levels with approximately 20 measurements of each. The thresholds for acceptance criteria of imprecision are usually set at a 20% coefficient of variation for immunoassays or MS methods; however, that threshold can be made stringent or slightly looser, depending on the LOQ, analyte, instrumentation, technique, and other factors (51, 52). Accuracy is determined by spike/recovery experiments and, whenever possible, comparison with other methods (53). The analytical measuring range and clinical reportable range with dilution validation are also defined at this stage (54), as well as the reference intervals (55), analytical specificity or selectivity with testing of common/probable interferences, sample stability, and preanalytical or instrument-specific experiments (e.g., carryover determination). It is important to say that most of these criteria are not unique to MS-based assays and many are also valid for immunoassay-based test development. Guidelines with MS-specific requirements have been created and widely adopted (50, 52, 56). However, development teams working on MAb assays by MS may often refer to guidelines for immunoassays, toxicology, and chemistry because of different preanalytical enrichment/extraction processes, unique potential interfering factors (e.g., endogenous human Ig) compared to small molecules. The complete validation of a new MAb LDT is approved by the laboratory management team and laboratory director. Best practices also suggest that peers who have not participated in the development process review all experiments and provide written feedback before a validation is accepted.

After validation meets all acceptance criteria, the method would then move into the clinical laboratory setting for the implementation stage. In implementation, the laboratory performs a preliminary evaluation to determine if the method performs to the validated specifications. The laboratory then performs verification studies to certify that the method is robust by performing experiments that test precision and accuracy, checking the analytical measuring range, reference intervals, and specified detection capability, at a minimum. If all experiments fall within planned acceptance criteria, the laboratory would be able to move forward with method implementation. The entire process may take up to 2 years, as it includes several information technology components and stamps of approval of regulatory agencies, such as documentation of the approved validation, setup of proficiency testing, and quality control programs to be accredited by the College of American Pathologists. In addition, both the laboratory director and the institution must hold specific licenses for testing of high complexity to offer the test to customers in New York State (NYS). In any new facility or test category, the laboratory has to pass an inspection by NYS auditors before testing is offered to patients. Therefore, the undertaking of bringing up new MAb testing is not small; the test sample volume is often taken into consideration by the clinical laboratory, as well as costs of sending out test samples to a reference laboratory, the cost of development, and potential revenue estimates. The decision of which platforms to choose and which tests to bring up will vary, depending on the laboratory's overall equipment list, expertise, other tests, and size.

## TRYPTIC PEPTIDE METHODS

For MAbs to be quantified by MS, they first have to be differentiated from the very similar polyclonal background of >1 g/dl of endogenous human Igs in serum. Historically, proteins have been quantitated by LC-MS/MS by using specific tryptic fragments (referred to as “proteotypic” peptides) (57). Multiple tryptic peptides can be quantitated at the same time, as shown for the quantitation of IgG subclasses in serum by LC-MS/MS (58), which used subclass-specific tryptic peptides from the constant region of each subclass heavy chain along with a common IgG tryptic peptide. For chimeric MAbs such as IFX and RTX, trypsin digestion is possible, as the nonhuman variable region is large (>250 amino acids) and the likelihood of finding unique signature peptides on the light chain and/or heavy chain that are specific to that MAb and not found in the human polyclonal background is the greatest (Table 2).

One of the challenges of the development of a tryptic method for large proteins such as MAbs is standardization of the digest. Most clinical MS assays are methods for small molecules that utilize a SIL IS that is added to the serum before extraction, which corrects for loss of analyte because of sample preparation and MS response. A clinically available tryptic method for quantitation of IFX utilizes a surrogate IS from a different species (horse) since a SIL version of IFX was not available at the time of development and the ones available today are prohibitively expensive. Horse IgG is added to patient serum before Ig enrichment by ammonium sulfate protein crash to monitor digestion efficiency. The Ig fraction from the ammonium sulfate crash is treated with trifluoroethanol, dithiothreitol (DTT), and iodoacetamide to unfold the protein, reduce the disulfide bonds between the heavy and light chains, and prevent their reformation (Fig. 1A). Trypsin is then added to cut the chains into specific peptides, two of these peptides being the IFX unique peptides from the light- and heavy-chain variable regions of the MAb (Fig. 1A). After the addition of isotopically labeled peptide retention time standards, the mixture is subjected to reverse-phase C_8_ LC and selective reaction monitoring with a triple-quadrupole mass spectrometer. Fragment ions from the IFX-specific peptides are monitored and compared to a standard curve for quantitation (59).

While the digest method may be the method of choice for some MAbs, not every MAb will be amenable to the digest method, especially as newer MAbs are engineered as humanized (ECU) or even fully human (ADM) (Table 2). Experiments have been performed to find a peptide specific for ADM, which is not found regularly in the polyclonal serum background; none was found (data not published). For other MAbs, the sensitivity of the method will be the limiting factor. For example, while RTX is a chimeric IgG1 MAb similar to IFX, it was found that the published tryptic method did not allow for the sensitivity needed for a clinical assay, but more specific, elaborate extraction methods may allow for enhanced sensitivity. ECU was also evaluated by the peptide method. As with RTX, it was also difficult to find a peptide that was unique to ECU and not common in the polyclonal background. Lastly, some laboratories may find the digest method referenced labor-intensive, since it consists of 2 days of manual analytical preparation and requires specialized personnel for pre- and postanalytical steps; it also requires expensive instrumentation.

## INTACT LIGHT CHAIN METHODS

**FIG 2:**
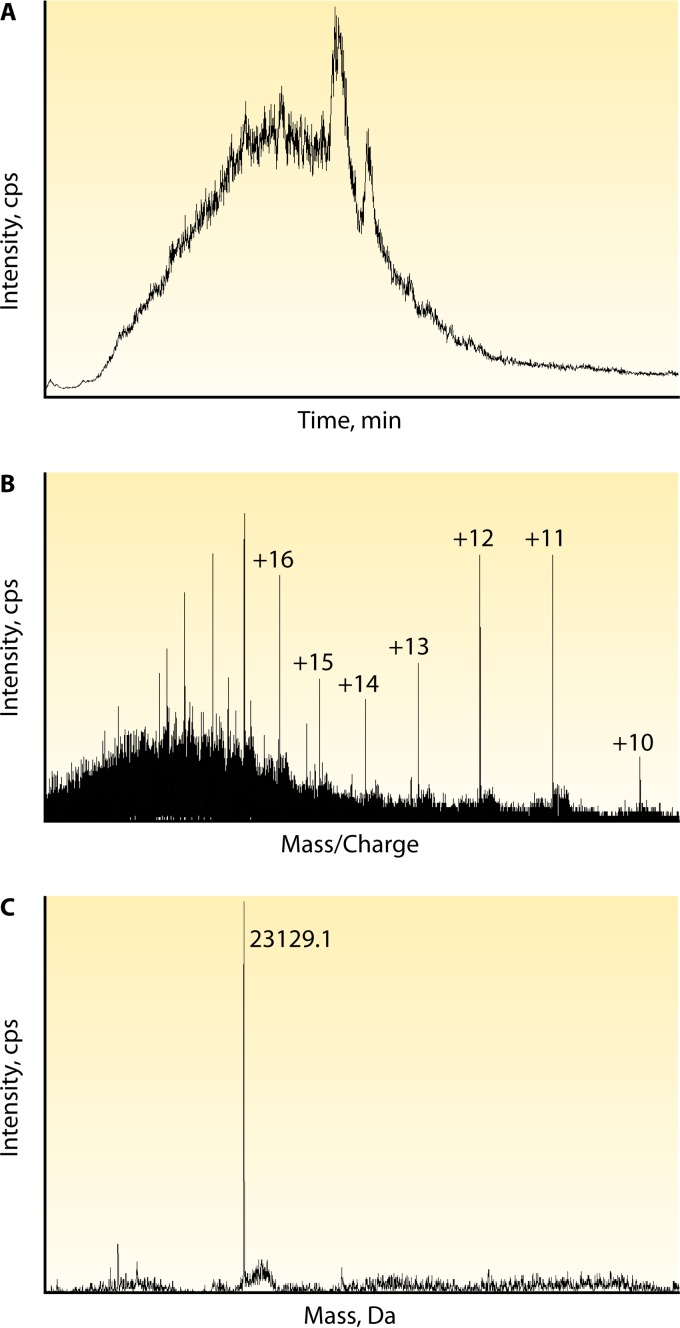
Data overview of LC-MS for miRAMM. The analysis of intact light chains by MS may be visualized in different ways. In this example, ECU at 100 μg/ml was used to spike normal human serum enriched for Ig with Melon Gel. (A) The total ion chromatogram from which a known MAb can be extracted. Light chains elute in a 5- to 8-min retention time window with a gradient mobile phase chromatographic protocol (9). (B) Once the retention time window is selected, the extracted ion spectrum shows all of the proteins eluted in that time frame, peak intensities, and the multiple charge states acquired. Visualization and quantitation are best at the +12, +11, and +10 charge states for Ig light chains by the published method. Depending on the software used, one or more charge states can be used for quantitation. (C) Deconvolution software will reconstruct the intact accurate mass from the acquired multiple charge states and may be used for MAb confirmation or quantitation.

In 2014, a method that used microLC-ESI-Q (quadrupole)-TOF MS, also referred to as miRAMM (monoclonal Ig rapid accurate mass measurement), was used to quantify intact kappa light chains from the therapeutic MAb ADM used to spike normal human serum. In that study, the therapeutic MAb was used as the model system to identify an endogenous monoclonal Ig in patients with multiple myeloma (60). This method was then adapted for utilization for the quantitation of the therapeutic MAb RTX in patients being treated for vasculitis (61). Quantification of MAb by measurement of its intact light chain mass utilizes Melon Gel (Thermo-Fisher Scientific, Waltham, MA) for extraction, an inexpensive, simple, and fast method to enrich a serum sample for Igs. The supernatant of the Melon Gel-purified mixture is reduced with DTT for 30 min and then analyzed by miRAMM (Fig. 1B). While full scan data are collected, the intact light chain mass of a particular MAb or one of the charge states can be monitored and used for quantitation against a standard curve of the pharmaceutical MAb used to spike pooled serum (Fig. 2).

While this method has been found to be amenable to MAbs that are not ideal for the peptide method, there are still limitations. For example, the intact method was found again to not be adequate for ADM, as trough MAb levels in patients undergoing therapy were lower than the LOQ of the assay, which made it unsuitable for routine use in the clinic for that specific analyte (data not published) (Table 2). Furthermore, the Melon Gel removes non-Igs from solution as its means of Ig enrichment. Therefore, it is possible that the MAb target is removed, and if it is complexed to the MAb, the Melon Gel extraction may not be able to recover the bound MAb-target complexes, resulting in underquantitation.

**FIG 3:**
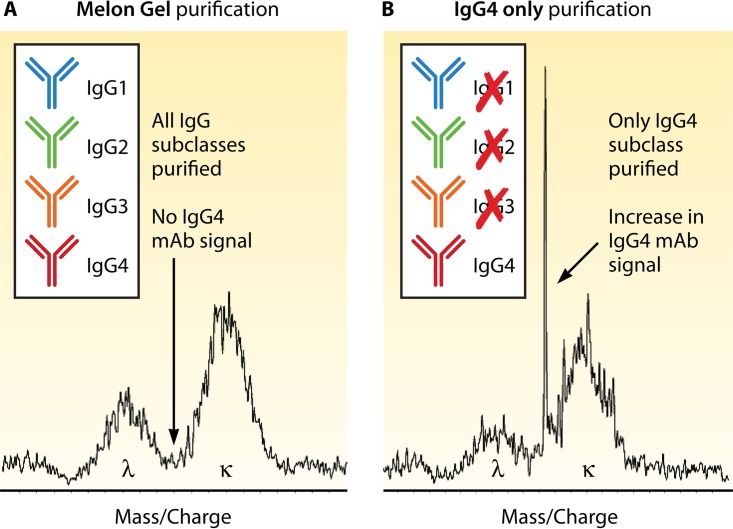
Ig enrichment strategies impact the LOQ. Observation of a unique MAb accurate mass in a serum matrix of patients undergoing therapy is accomplished among 1 g/dl of human endogenous circulating Igs. Depending on the MAb molecular mass, the LOQ can be an issue, as shown in this miRAMM example. When studying ECU, an IgG2/IgG4 hybrid MAb, different enrichment strategies were employed. (A) When Melon Gel enrichment was used, an ECU concentration of 5 μg/ml was not detectable. (B) Selective enrichment for IgG4 with an affinity matrix allowed for significant reduction of the endogenous Ig repertoire by removing all non-IgG4 Ig from the background and increased the analytical sensitivity of the assay by approximately 10-fold, with an increased signal-to-noise ratio.

These limitations have led to the exploration of new extraction techniques specifically, as the factor limiting sensitivity is distinguishing a therapeutic MAb from the patient's normal endogenous polyclonal Ig background. Most therapeutic MAbs have an IgG heavy chain and a kappa light chain. IgG antibodies make up 75% of the serum antibodies and are categorized into four different subclasses. The subclass with the lowest concentration is IgG4, which makes up only about 4% of the total IgG (62). The ability to selectively extract only the IgG4 antibodies from serum should theoretically allow for an advantage in detection and simplify the therapeutic monitoring of IgG4 MAb therapies. ECU, as an IgG2/4 hybrid, was perfect for the exploration of this new extraction method. By utilizing CaptureSelect IgG4(Hu) Affinity Matrix (Life Technologies, Carlsbad, CA), a matrix that enriches for only the IgG4 subclass instead of utilizing Melon Gel, which enriches for all IgGs, a 10-fold increase in sensitivity was gained for ECU (Fig. 3) (63).

**FIG 4:**
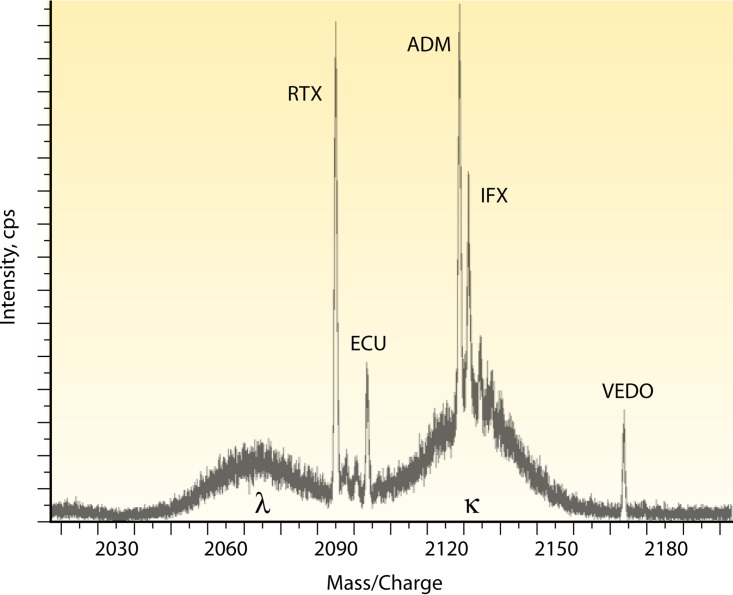
Therapeutic MAbs can be measured simultaneously by miRAMM. In this example, several therapeutic MAbs were used to spike commercially available normal human serum. A given therapeutic MAb's specific light chain will have a unique mass and be distinguishable from the endogenous Ig background when present in large concentrations. The extracted ion spectrum shows the +11 charge state of five MAbs' specific light chains detected above the human serum polyclonal Ig background. Although it is highly unlikely that a patient would not be undergoing therapy with five or six different MAbs at the same time, the multiplex capability allows application of the technique for screening methods, such as the differentiation of a therapeutic MAb from a disease-causing endogenous monoclonal protein.

The LC-MS miRAMM method has proved to be a versatile technique, allowing the quantification of multiple MAbs simultaneously, which would allow for a multiplex quantitation method, a screening method, or the possibility of both. The power of the screening method can be seen when five MAbs are mixed together and diluted in commercial grade normal human serum. Figure 4 shows the +11 charge state for each of the five MAbs and where each elutes in mass space with the polyclonal serum background. The innovative aspect of this methodology is that it not only quantifies therapeutic MAbs but can also provide information on a patient's polyclonal phenotype response during treatment, all in the same analytical run. In addition, the technique does not rely on using the target antigen as part of the assay. Our group is currently using this methodology to quantify MAbs in clinical studies. Our preliminary findings to date show that the concentrations found by microLC-ESI-Q-TOF MS are in excellent agreement with those found by non-MS-based methods (data not shown).

## FUTURE DIRECTIONS

Although there are currently only a few MAbs with indications of a companion clinical assay for therapeutic efficacy monitoring, the use of drug quantitation and the assessment of immunogenicity in patient care will only continue to expand, as should our understanding of their impact on routine laboratory testing. Although there are many challenges, this field presents another opportunity for the clinical laboratory to impact patient management and improve outcomes for our patients.

The use of SIL ISs is the best practice in toxicology and endocrinology, now that commercial laboratories can readily synthesize small organic analyte molecules and peptides incorporating heavy isotopes (i.e., C_13_, N_15_) for clinical laboratories to use as an IS. An IS is added to a sample to compensate for imprecision in the extraction protocol and LC retention times since it behaves exactly the same as the native analyte but its molecular mass is different and therefore it can be quantified separately from the native analyte. The ratio of the abundance of the native analyte to the abundance of the IS serves as the value compared to a standard curve for quantification. Synthesizing an intact MAb incorporating SIL amino acids is a substantial challenge since it is much larger than a peptide (>100-fold) and must be expressed in a cell culture system, adding substantial cost, thus significantly limiting its use. The lack of SIL has forced mass spectrometrists to use alternative ISs in proteomics, which include the use of other MAbs with similar characteristics or animal polyclonal Igs. The pharmaceutical industry has been searching for a universal SIL to be used in analytical measurements and although commercial products have become available (e.g., SILuMAb [Sigma-Aldrich]), they are far from being a “one size fits all” approach and also have limitations, such as a different structure, cleavage sites for trypsin, PTMs, and retention times different from that of the target MAb.

New generations of digest methods are under development. The *in vitro* diagnostics industry is offering digest kits that would streamline the development and implementation of MAb assays, since optimization studies of reduction agents and trypsin incubation are then performed by the manufacturers, and the preweighed reagents, lot traceability, and quality standards allow for a shorter yet efficient digest and the possibility of converting a long manual assay and making it automatable.

**FIG 5:**
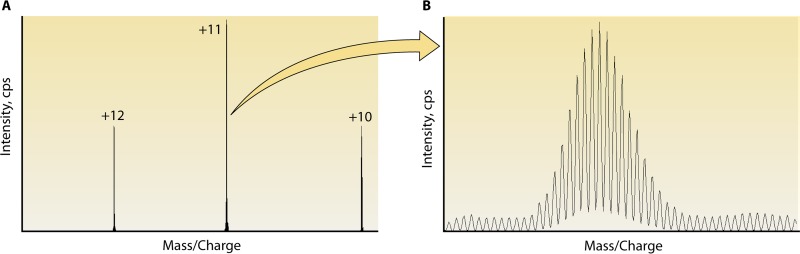
Multiple charge states acquired by high-resolution MS for ECU. Normal human serum was spiked with ECU at 100 μg/ml and enriched for Ig with Melon Gel. After ESI, the light chains acquired multiple charge states and were detected with an Orbitrap (Thermo Fisher) instrument. (A) Mass spectrum of the +12, +11, and +10 charge states. Utilizing low-resolution scanning, the areas under the curve of one or multiple charge states are added together for quantitation of the MAb. By using a high-resolution view, the area under the curve of multiple isotopes of a given charge state can be analyzed. (B) Isotopic distribution of a +11 charge state.

In an effort to increase sensitivity, instruments such as the Q Exactive, which has a quadrupole connected to an Orbitrap mass spectrometer, are being evaluated. The Q Exactive has the capacity to scan small targeted mass/charge windows (referred to as a t-SIM scan) at extremely high resolution (140,000), enabling the isotopes of each MAb charge state to be analyzed and used for quantification. It is worth repeating that an instrument with such high resolution has not been available to clinical labs until recently. As a result, adding together the peak areas of single isotopes from multiple charge states is a radically new approach to MAb quantification. Furthermore, the Q Exactive can perform multiple scans simultaneously, a low-resolution full scan for evaluating endogenous Igs plus a high-resolution targeted scan for MAb quantitation (Fig. 5). Combined, these attributes make the Q Exactive Orbitrap mass spectrometer an exciting new option for clinical chemists and immunologists interested in MAb quantification.

Recently, several therapeutic MAbs came off patent and biosimilars have become available. Biosimilars are MAbs based on the original product, with identical amino acid sequences and proven therapeutic equivalence, but the manufacturing process may occur in different cell culture systems. In 2016, the FDA approved biosimilar MAbs for IFX and ADM. The competition for market share is becoming more aggressive. Companies are investing resources into offering serum laboratory measurements to patients on the original MAb as a benefit, to dissuade them from switching to biosimilars. Versatile assays capable of measuring both the original and biosimilar MAbs will have an advantage, and we predict that optimizing therapeutic regimens and monitoring loss of response to therapy will become the standard of care as patients start to have more options of MAbs to treat diseases previously thought untreatable.

## CONCLUSION

Currently, there are a limited number of commercially available clinical assays for quantifying therapeutic MAbs. Considering the double-digit growth rate of therapeutic MAbs, there will likely be a need for more clinical assays to quantify them in the near future. Historically, clinical assays have relied on ELISAs to quantify MAbs. However, as we have presented here, MS-based clinical assays are an entirely new approach to therapeutic MAb monitoring that has distinct advantages over ELISAs. LC-MS/MS methods of MAb quantification performed on a triple-quadrupole mass spectrometer are already in production, with additional LC-MS-based assays performed on TOF and quadrupole-Orbitrap mass spectrometers nearing validation. The success of therapeutic MAbs such as DARA and PEMBRO in clinical oncology trials granted them accelerated FDA approval status. This is evidence of the potential that therapeutic MAbs have to significantly improve the quality of life of patients with a diverse array of conditions. Therapeutic MAbs also have the potential to revolutionize what we know about our immune system. Laboratorians will not be left out of this revolution and will likely have to reinvent the way routine immunoassays are performed for patients undergoing MAb therapy. In our view, MS-based assays will likely play a major role in the quantification of this unique class of drugs.
